# Electrofusion of Mesenchymal Stem Cells and Islet Cells for Diabetes Therapy: A Rat Model

**DOI:** 10.1371/journal.pone.0064499

**Published:** 2013-05-28

**Authors:** Goichi Yanai, Takashi Hayashi, Qi Zhi, Kai-Chiang Yang, Yasumasa Shirouzu, Takashi Shimabukuro, Akihito Hiura, Kazutomo Inoue, Shoichiro Sumi

**Affiliations:** 1 Department of Organ Reconstruction, Institute for Frontier Medical Sciences, Kyoto University, Kyoto, Japan; 2 Gakkentoshi Hospital, Kyoto, Japan; 3 Department of Histology and Embryology, School of Medicine, Nankai University, Tianjin, China; 4 School of Dentistry, College of Oral Medicine, Taipei Medical University, Taipei, Taiwan; 5 Takeda Bio Health Research Center, Kyoto, Japan; 6 Inoue Clinic Diabetes Center, Kyoto, Japan; University of Bremen, Germany

## Abstract

Islet transplantation is a minimally invasive treatment for severe diabetes. However, it often requires multiple donors to accomplish insulin-independence and the long-term results are not yet satisfying. Therefore, novel ways to overcome these problems have been explored. Isolated islets are fragile and susceptible to pro-apoptotic factors and poorly proliferative. In contrast, mesenchymal stem cells (MSCs) are highly proliferative, anti-apoptotic and pluripotent to differentiate toward various cell types, promote angiogenesis and modulate inflammation, thereby studied as an enhancer of islet function and engraftment. Electrofusion is an efficient method of cell fusion and nuclear reprogramming occurs in hybrid cells between different cell types. Therefore, we hypothesized that electrofusion between MSC and islet cells may yield robust islet cells for diabetes therapy. We establish a method of electrofusion between dispersed islet cells and MSCs in rats. The fusion cells maintained glucose-responsive insulin release for 20 days in vitro. Renal subcapsular transplantation of fusion cells prepared from suboptimal islet mass (1,000 islets) that did not correct hyperglycemia even if co-transplanted with MSCs, caused slow but consistent lowering of blood glucose with significant weight gain within the observation period in streptozotocin-induced diabetic rats. In the fusion cells between rat islet cells and mouse MSCs, RT-PCR showed new expression of both rat MSC-related genes and mouse β-cell-related genes, indicating bidirectional reprogramming of both β-cell and MSCs nuclei. Moreover, decreased caspase3 expression and new expression of Ki-67 in the islet cell nuclei suggested alleviated apoptosis and gain of proliferative capability, respectively. These results show that electrofusion between MSCs and islet cells yield special cells with β-cell function and robustness of MSCs and seems feasible for novel therapeutic strategy for diabetes mellitus.

## Introduction

Diabetes mellitus (DM) is a leading cause of morbidity and mortality in industrialized countries, and the number of patients affected is estimated to be 366 million in 2011 with an increase to 552 million by 2030 [1]. Among several types of DM, Type 1 DM (T1DM) is characterized by the selective destruction of pancreatic β-cells caused by an autoimmune attack or other unknown causes. β-cell reconstruction is currently achieved only by either pancreas or islet transplantation in clinical setting. Although clinical trials of encapsulated islets that enable transplantation without immune suppression are on-going [2], these transplantation therapies share common problems of donor scarcity and adverse effects related to immune suppression.

Islet transplantation is an effective therapy for T1DM, but limited donor sources restrict it from becoming a major treatment option [3], [4]. In islet transplantation, a diabetic patient often requires two or even three donor pancreata to accomplish insulin-independence in current mainstream protocols, which makes the problem of a donor shortage even more serious [5]. Even though insulin-independence is achieved by islet transplantation, islet graft function is rarely sustained with only 7.5% of these patients remaining insulin-independent at 5 years post transplantation [3].

Loss of functional isolated islets occurs during the culture period after isolation and purification [6]. It is established that apoptosis triggered by withdrawal of growth factors [7], disruption of extracellular matrix [6], [8], and endotoxin contamination [9] participates in islet loss under culture conditions. From these reports, β-cells in isolated islets are susceptible to immune and inflammatory factors and have minimal proliferation capacity, if any.

Mesenchymal stem cells (MSCs), which were first identified by Friedenstein and his colleagues [10], are known to be highly proliferative and with anti-apoptotic potential [11]. MSCs derived from bone marrow and other organs such as liver, umbilical cord blood, placenta, and adipose tissue [12]–[15] have high proliferation capacity and multipotency to differentiate toward various cell types such as muscle, cartilage, and bone [16]. In addition, MSCs have been shown to promote angiogenesis *in vivo* [17], [18].

Recent studies have shown that MSCs secrete several factors that improve survival and function of transplanted islets. MSCs co-cultured with islets secrete higher levels of anti-apoptotic signaling molecules and improve glucose-stimulated insulin secretion indexes [19]. Bone marrow cells are also shown to induce endogenous β-cell proliferation and improvement of islet function *in vivo* [20]. Furthermore, in co-transplantation of MSCs with islets, MSCs improved the capacity of islet grafts to reverse hyperglycemia compared with islets alone [21]. MSCs may also enhance islet resistance to hypoxia/reoxygenation-induced apoptosis and dysfunction by promoting anti-apoptotic gene expression [22]. Because of these favorable effects on islets, MSCs provide an important approach for improvement of islet engraftment, thereby decreasing the numbers of islets needed to achieve insulin-independence [23].

Cell fusion occurs in physiological conditions such as normal development and immune reaction [24]–[26]. Bone marrow-derived stem cells fuse to several types of cells under normal condition or after cell injury [27] and Tada M et al. have shown that the nuclei of somatic cells can be reprogrammed by cell fusion with embryonic stem cells [28]. Therefore, cellular transformation may be induced by cell fusion between different types of cells through nuclear reprogramming.

On the basis of above-mentioned knowledge, it was hypothesized that cell fusion between MSC and β-cells may produce a novel type of cells that combines β-cell function with MSC characteristics including proliferation capacity and anti-apoptotic ability.

In this study, we established a method of electrofusion between MSCs and pancreatic islet cells and examined insulin secretion capacity, mutual nuclear reprogramming, anti-apoptotic and proliferative changes of islet cell nuclei *in vitro* and verified the potential application of fusion cells to regenerative medicine for diabetes mellitus *in vivo*.

## Materials and Methods

### Animals

Rats and mice were purchased from Shimizu Laboratory Supplies Co. Ltd. (Kyoto, Japan). These animals were housed in climate-controlled rooms with free access to pellet food and water. The approval to conduct this experiment was obtained from the Animal Care Committee of Institute for Frontier Medical Sciences, Kyoto University, and the animals were treated according to the experimental protocols under its regulations.

### Reverse Transcription Polymerase Chain Reaction (RT-PCR)

In the present study, total RNA was extracted using PureLink RNA Mini kit (Invitrogen). RT-PCR was carried out using a SuperScriptIII First-Strand cDNA synthesis (Invitrogen) and a thermal cycler, iCycler (Bio-Rad). The primers used for PCR are shown in the tables designated for each experiment. Unless otherwise noted, PCR were performed for 35 cycles with each cycle comprising 20 sec at 94°C, 30 sec at 65°C, and 1 min at 72°C. A final cycle comprised 5 min at 72°C. After PCR, electrophoresis was performed by E-Gel iBase Power System and E-Gel 4% Agarose (Invitrogen).

### Preparation of MSCs

The bone marrow was isolated from the tibias and femurs of Lewis rats (male, 5 weeks old) or C57/BL6 mice (male, 4 weeks old). After washing 3 times with Hank’s balanced salt solution and centrifugation (1,000 rpm for 5 min at 4°C), cells were plated to 225 cm^2^ flask in the 1∶1 mixture of Dulbecco’s modified Eagle’s Medium and F12 (DMEM/F12; Gibco, NY, USA) with 12.5% fetal bovine serum (FBS) and 1% antibiotics solution (mixture of 100 units/mL penicillin G sodium, 100 µg/mL streptomycin sulfate and 25 µg/mL amphotericinB; Gibco) at 37°C in a humidified atmosphere of 5% CO_2_ and 95% air. Non-adherent cells were removed after 3-day culture. Adherent cells were detached with 0.5% Trypsin-EDTA (Gibco) when the cells become confluent after 4- to 5-day culture. MSCs of passages 5–10 were used in the following experiments.

To characteraize MSCs, the cells were examined by RT-PCR about four genes, i.e., CD34 and CD45 as negative markers and CD73 and CD105 as positive markers. Bone marrow was served as the control for MSCs. Primers are shown in Table 1.

**Table 1:** Primer sequence for RT-PCR.

Animal	Gene		
Rat	CD34	Forward	AGCCATGTGCTCACACATCA
		Reverse	CAAACACTCGGGCCTAACCT
	CD45	Forward	TTGCTCCCCATCCGATAAGAC
		Reverse	AGCGTGGATGAAAAACCATCG
	CD73	Forward	TGCATCGATATGGCCAGTCC
		Reverse	AATCCATCCCCACCGTTGAC
	CD105	Forward	ACTGAGTTGCACATCTGGGG
		Reverse	TTCCGAAGTGGTGGTAAGCC
Mouse	CD34	Forward	CAGGAGAAAGGCTGGGTGAA
		Reverse	GTTGTCTTGCTGAATGGCCG
	CD45	Forward	ACTGAATCCACACCCCCAAG
		Reverse	AGCTTGGCTGCTGAATGTCT
	CD73	Forward	AGTTCTCTCTGTTGGCGGTG
		Reverse	GGATGCCACCTCCGTTTACA
	CD105	Forward	TGTACCCACAAGTCTCGCAG
		Reverse	ATGCTTTGGGGGTCATCCAG

### Islet Isolation

Islets were isolated from male Lewis rats (11 weeks old, 280–300 g) as described previously [29]. Briefly, rat pancreata were digested by collagenase (typeXI, Sigma, St. Louis, USA) and then the islets were separated by a dextran gradient. The islets were further purified by handpicking and then were cultured in CMRL-1066 medium (Gibco) with 10% FBS and antibiotics at 37°C in a humidified atmosphere of 5% CO_2_ and 95% air. After 24-hour culture, the islets were treated with 0.5% Trypsin-EDTA for 10 min at 37°C in order to prepare dispersed islet cells. Then, dispersed cells were collected by centrifugation (1200 rpm, 5 min at 4°C) and temporarily kept in the same medium at 37°C until used for experiment within 20 min.

### Cell Fusion

MSCs and dispersed islet cells were washed once in fusion medium containing 5% glucose, 0.1 mM CaCH_3_(COO)_2_, 0.5 mM MgCH_3_(COO)_2_ and 0.3% bovine serum albumin (BSA). The pH of the fusion medium was adjusted to 7.2–7.4 with L -histidine (all chemicals were from Sigma). After centrifugation, the cells were re-suspended in the fusion medium without BSA. MSCs (1×10^6^) and dispersed islet cells (1×10^6^) were suspended in 100 µl of fusion medium and placed in a specially designed fusion chamber made of two concentric oval electrodes (6c m×16 cm, Cat. No. CUY480G2, NEPA GENE Co., Ltd. Chiba, JAPAN). For electrofusion, a pulse generator (ECM 2001, BTX Instrument, Genetronics, CA, USA) was used. Electrofusion involved two independent but consecutive steps. The first treatment is to bring cells in close contact by dielectrophoresis, which can be accomplished by exposing cells to an alternating electric field (AC) of relatively low voltage. Then, cell fusion was triggered by applying a single squarewave pulse (DC) to induce reversible cell membrane break-down in the zone of membrane contact. For this study, electrofusion was perfomed by AC of 35 V for 20 sec followed by DC of 350 V for 25 µsec based on our past study [30].

### Validation of Cell Fusion

To determine fusion efficiency, cell mixtures before cell fusion and fusion cells were stained by Giemsa staining after 24 hour culture. In order to confirm cell fusion between MSCs and islet-cells, MSCs and dispersed islet cells were pre-labeled with the SYTO11 (Invitrogen, CA, USA) and Vybrant Dil (Santa Cruz, CA, USA), respectively, following manufacturer’s instructions. Cells before and after cell fusion were examined under a fluorescence microscope (Olympus IX70, Tokyo, Japan) after an overnight culture.

### Insulin Secretion Test

*In vitro* glucose challenge test was performed in the prepared cells as follows after 1-, 10- and 20-day culture: (1) MSCs only (2×10^4^ cells per well), (2) Islets only (20 Islets), (3) Non-fused MSCs (2×10^4^ cells) with islets (20 islets), (4) Non-fused MSCs (2×10^4^ cells) with dispersed islet cells prepared from 20 islets, (5) Fusion cells of MSCs (2×10^4^ cells) and dispersed islet cells prepared from 20 islets. For glucose challenge test, all groups were pre-incubated in RPMI-1640 with 0.1% BSA containing 3.3 mM glucose at 37°C for 1 hour. After pre-incubation, the medium was replaced with the same medium for 1 hour. Then, the medium was replaced with RPMI-1640 with 0.1% BSA containing 16.7 mM glucose for 1 hour. Finally, the medium was replaced with RPMI-1640 with 0.1% BSA containing 3.3 mM glucose for 1 hour. Insulin concentration of the media was measured using a rat insulin ELISA kit (Shibayagi, Gunma, Japan).

### Nuclear Reprogramming

In order to investigate whether nuclear reprogramming occurs in MSCs and/or islet cells, mouse MSCs and rat islet cells were fused and expressions of typical MSC genes (Oct3/4, CD106, and Sca1) and islet genes (Insulin-1, Pdx-1 and Ngn3) were examined by RT-PCR after 1-day culture using the primers designed for both rat and mouse genes.

Total RNA was extracted from MSCs of mouse and rat, rat islets, MIN-6 cells [31] and the fusion cells. Co-culture of mouse MSCs with rat islets (MM+RI) was served as the control for fusion cells. The primers are shown in Table 2.

**Table 2:** Primer sequence for RT-PCR.

Animal	Gene		
Rat	Inslin-1	Forward	GAGGACCCGCAAGTGCCACA
		Reverse	GGCGGGGAGTGGTGGACTCA
	pdx1	Forward	TTGCAGGCTCGCTGGGAACG
		Reverse	AGCAGCTGGGCCCGAGTGTA
	Ngn3	Forward	TGGGCCCCCGTTGCTGATTG
		Reverse	ACGCGGGACTAGAGTACGCCC
	CD106	Forward	GGTGGCTGCACAGGTTGGGG
		Reverse	ACCCACAGGGCTCAGCGTCA
	sca1	Forward	TCTTTGCAACGCAGCAGGGC
		Reverse	CACGTGCCTCCAGGGCCAAG
	oct3/4	Forward	GGGGAGCCCACCTTCCCCAT
		Reverse	ACGGGGAGATCCCCAGCACC
	β-actin	Forward	GCGAGTACAACCTTCTTGCAGCTC
		Reverse	TGGCATGAGGGAGCGCGTAA
Mouse	Inslin-1	Forward	TGGAGCTGGGAGGAAGCCCC
		Reverse	ATTGCAAAGGGGTGGGGCGG
	pdx1	Forward	CAAAGCGATCTGGGGTGGCGT
		Reverse	CGCTGAACTCTGGCACCGGG
	Ngn3	Forward	TGCCCGCTACATGCAGGGTT
		Reverse	AGGAACCGTCCCTGCAACTCAC
	CD106	Forward	CGTGGGGACTTGGCTGGCTG
		Reverse	AGCCGGGCTGGTGTGAGTGA
	sca1	Forward	GCCCCTGCTGGGTAGGTAGGT
		Reverse	TGTGCTGGCTGTGTGCCTCC
	oct3/4	Forward	CTGCCCCCAGGTCCCCACTT
		Reverse	AGCATCCCCAGGGAGGGCTG
	β-actin	Forward	AGGCGGACTGTTACTGAGCTGC
		Reverse	CTCAGGGCATGGACGCGACC

### Islet Cell Apoptosis

In order to investigate whether electrofusion of rat MSCs induce changes on rat islet cell apoptosis, rat caspase3 gene expression was examined by RT-PCR in five groups described in insulin secretion test after 1-day culture. The primers used for PCR are shown in Table 3. PCR were performed for 28, 30, 32, 34 and 36 cycles with each cycle comprising 10 sec at 98°C, 30 sec at 65°C, and 1 min at 72°C. A final cycle comprised 5 min at 72°C. After PCR, electrophoresis was performed by E-Gel iBase Power System and E-Gel 4% Agarose (Invitrogen). Moreover, in order to detect apoptotic cells, all groups were stained with annexin V and propidium iodide (PI) using Annexin V-FITC (Beckman Coulter, Tokyo, Japan, IM2375) following manufacturer’s instructions. Briefly, cell samples were suspend in accompanying buffer on ice. FITC-labeled annexin V solution and PI solution were added and the samples were kept on ice for 10 minutes in the dark. Then, the samples were observed under a fluorescence microscope (KEYENCE BZ-8000, Tokyo, Japan).

**Table 3:** Primer sequence for RT-PCR.

Animal	Gene		
Rat	caspase3	Forward	CTTTGCGCCATGCTGAAACT
		Reverse	ATGACGACCTGGAACATCGG

### Islet Cell Proliferation

In order to investigate whether islet cell proliferation occurs in fusion cells, fusion cells were made from mouse MSCs and rat islet cells. Expressions of rat Ki-67 gene was examined by RT-PCR using the primers shown in Table 4 after 1- and 5-day culture. Mouse MCSs, rat MSCs, rat islets and co-culture of mouse MSCs with rat islets were served as controls.

**Table 4:** Primer sequence for RT-PCR.

Animal	Gene		
Rat	Ki-67	Forward	CTTTGCGCCATGCTGAAACT
		Reverse	ATGACGACCTGGAACATCGG

### Transplantation Experiment

In order to investigate whether fusion cells can really control hyperglycemia, the effect of fusion cell transplantation was observed in comparison to optimal (2,000 islet per rat) and suboptimal (1,000 islet per rat) islet mass transplantation. Lewis rats (male, 11 weeks old, 280–300 g) were made diabetic with streptozotocin (55 mg/kg body weight, i.p.) for recipients. One week after the injection, blood glucose (BG) was measured by tail vein sampling using Fuji Dry Chem system (Dri-chem 3000 colorimetric analyzer, Fujifilm, Tokyo, Japan) and rats with non-fasting BG higher than 500 mg/dL were used for recipients. Rats were divided into seven groups as follows: Group1; Normal control group (n = 6), Group 2; sham operated DM group (n = 6), Group 3; sub-optimal islet group (1000 islets: n = 9), Group 4; optimal islet group (2000 islets: n = 5), Group 5; MSCs group (1×10^6^ cells: n = 9), Group 6; Non-fused cell group (dispersed islet cells from 1000 islets and MSCs 1×10^6^ cells: n = 9), Group 7; Fusion cell group (fusion cells processed from dispersed cells of 1000 islets and MSCs 1×10^6^ cells: n = 9). Cells were transplanted into the left renal subcapsular space through the skin incision on the lumbar dorsum under general anesthesia of isoflurane inhalation. After transplantation, BG and body weight were measured on postoperative days (POD) 3, 7, 14, 21, 28, 35, 42, 49, 56, 63, 70, 77, 84, and 91.

### Statistical Analysis

Results are presented as mean ± SE as indicated in the figure legends. Two-way ANOVA for repeated-measures in SPSS 14.0 for windows was used, and p<0.05 was chosen as the level of significance.

## Results

### Characterization of MSCs

**Figure 1:**
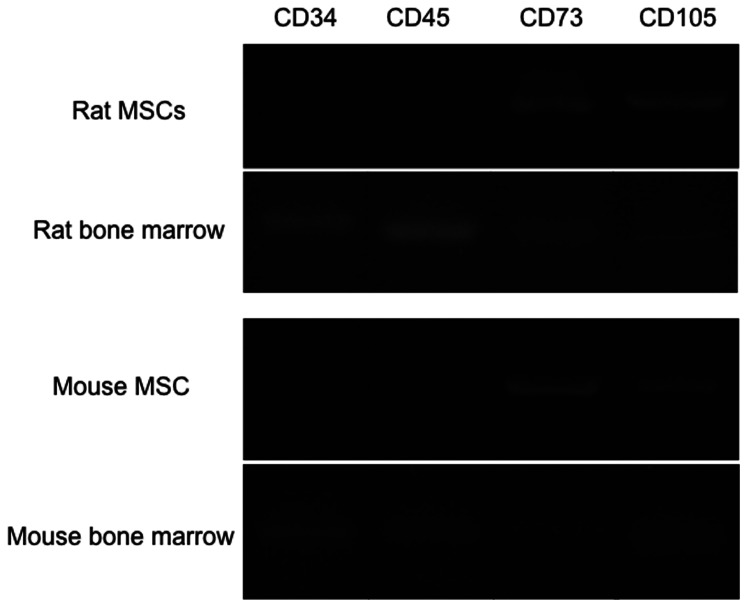
Gene expression of CD34, CD45, CD73 and CD105 in rat MSCs, rat bone marrow, mouse MSCs and mouse bone marrow.

**Figure 2:**
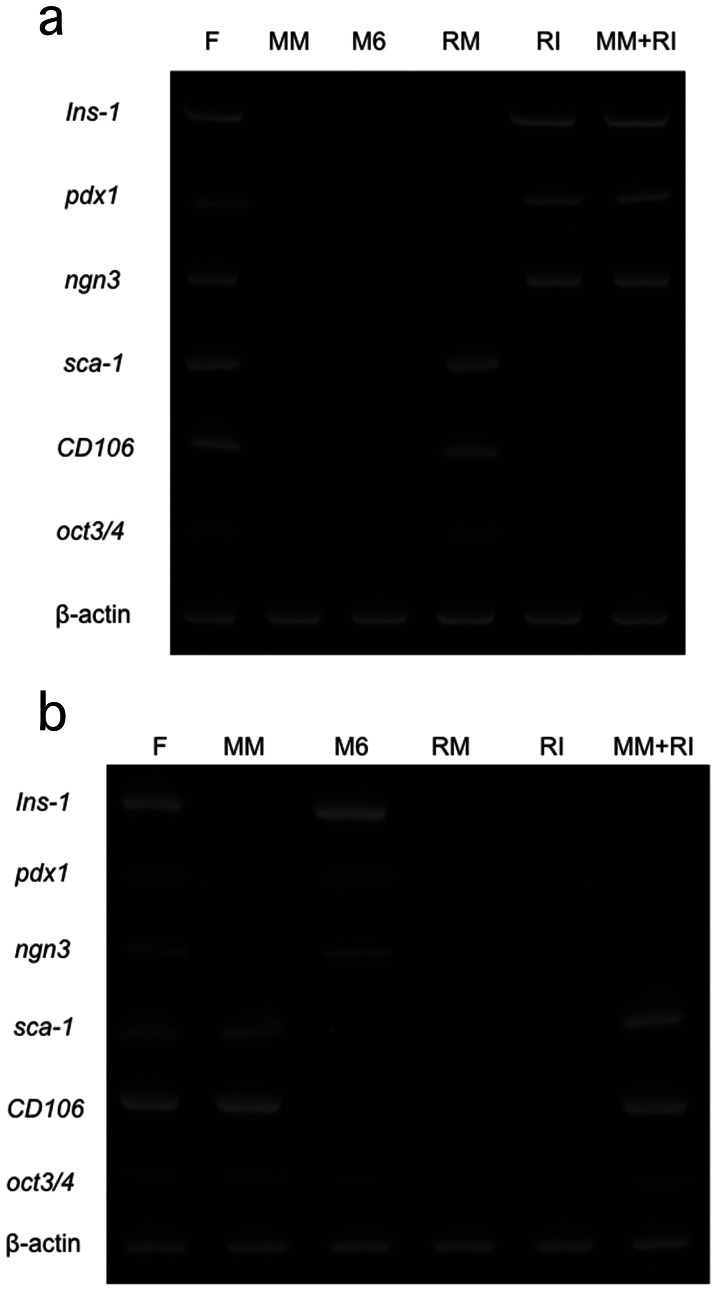
Results of reverse transcription PCR for reprogramming after 1-day culture. PCR was carried out by primers designed for rat genes (a) and mouse genes (b). F: Fusion cells between mouse MSCs and rat islet cells, MM: mouse MSCs, M6: MIN-6, RM: rat MSCs, RI: rat islets and MM+RI: co-culture of mouse MSCs with rat islets (non-fused).

MSCs have been established using various methods by many researchers. In the present study, our rat and mouse bone marrow-derived cells showed morphology similar to typical MSCs and expressed several MSC-specific markers such as CD73 and CD105 (Fig. 1) in addition to sca-1 and CD106 (Fig. 2-a, b). On the other hand, CD34 and CD45 that were expressed in original bone marrow cells were not detected (Fig. 1). These results indicate that our bone marrow-derived cells were consistent with putative MSCs.

### Validation of Cell Fusion

**Figure 3:**
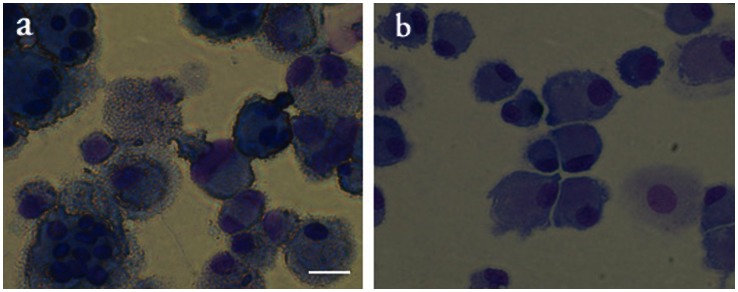
Giemsa staining in electrofusion cells (a) and non-fused cells (b). Scale bar: 20 µm.

**Figure 4:**
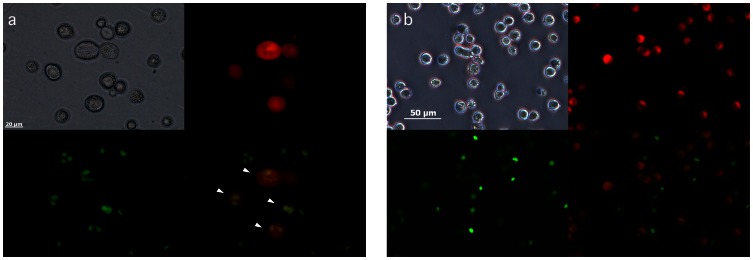
Fluorescence staining in electrofusion cells (a) and non-fused cells (b). Left upper panel shows light microscopic view. Islet-cells were stained by Vybrant-Dil (red: right upper panel), and MSCs were stained by SYTO11 (green: left lower panel). Right lower panel shows merge (arrow head: fusion cell). Fusion cells show yellowish nuclei since green nuclei are involved in red cytoplasm.

Rat MSCs and dispersed rat islet-cells were successfully fused by our method. By Giemsa staining, multinuclear cells were observed after cell fusion whereas such cells were not observed without cell fusion (Fig. 3). By electrofusion of SYTO11-labeled MSCs and Vybrant Dil-labeled islet cells, cells double positive for SYTO11 and Vybrant Dil were observed after cell fusion, whereas such cells were not observed without cell fusion (Fig. 4).

### Insulin Secretion Test

**Figure 5:**
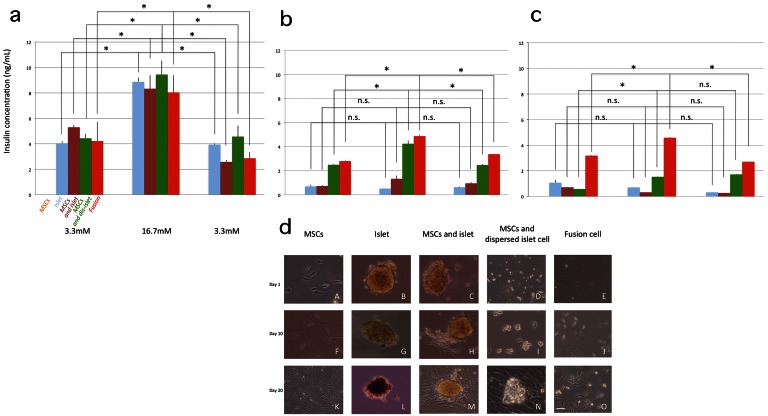
*In vitro* insulin secretion and microscopic morphology of the tested cells. a–c: insulin secretion after 1, 10, and 20 day cultures, respectively. (mean±SEM: n = 3, *:p<0.05). d: microscopic morphology, scale bar: 20 µm A–E: after 1 day culture. F–J: after 10 days of culture. K–O: after 20 days of culture.

In comparison among the cultured cells of MSCs alone, islets alone, MSCs and islets without cell fusion, MSCs and dispersed islet cells without cell fusion and fusion cells of MSCs and dispersed islet cells, all groups except for MSCs alone showed glucose-responsive insulin release after 1-day culture (Fig. 5-a). After 10-day culture, islets alone lost the glucose responsiveness but dispersed islet cells with MSCs and fusion cells maintained it (Fig. 5-b). Although undispersed islets with MSCs seemed to maintain responsiveness a, the insulin concentration was lower and the response was not statistically significant (Fig. 5-b). After 20-day culture, all groups except fusion cells showed loss of glucose-responsiveness (Fig. 5-c). In dispersed islet cells with MSCs, although significant increase was observed between the first 3.3 mM and 16.7 mM, decreased between 16.7 mM and the last 3.3 mM was not observed. Therefore, these cells were considered to have lost good glucose-responsiveness.

Figure 5-d shows microscopic morphology of the tested cells. Figure 5-d (A–E) shows the cells after 1-day culture, figure 5-d (F–J) shows those after 10-day culture and figure 5-d (K–O) shows cells after 20-day culture. In islets alone, most of the islets were destroyed after 20-day culture (Fig. 5-d: L). In the co-cultures of MSCs and islets, a few islets maintained their morphology and they were attached to colonies of MSCs (Fig. 5-d: M). In co-culture of MSCs and dispersed islet-cells, re-clustering of islet cells was observed although it is not known if such clusters contained MSCs (Fig. 5-d: N). In fusion cells, most cells were attached to the bottom of the culture dish and cell number appeared to increase with time (Fig. 5-d: E, J, O).

### Nuclear Reprogramming

MSC- and β-cell-related gene expression of fusion cells between mouse MSCs and rat islets was examined. Primers designed for rat genes (Table 2) did not react with the cDNA derived from mouse MSCs and MIN-6, a mouse β-cell line. Rat islet markers such as Insulin-1, Pdx-1 (Pancreatic duodenal homeobox 1), and Ngn3 (Neurogenin 3) were not expressed in rat MSCs and conversely, rat MSC markers such as Sca-1, CD106 and Oct3/4 were not expressed in rat islets. Fusion cells prepared from rat islets and mouse MSCs showed new expression of the rat MSC markers (Fig. 2-a). Similarly, primers designed for mouse genes (Table 2) did not react with the cDNA derived from rat islets and MSCs. Mouse islet markers were not expressed in mouse MSCs. Mouse MSC markers except for Oct3/4, were not expressed in MIN-6. Fusion cells showed new expression of the mouse islet markers (Fig. 2-b). On the other hand, such changes in gene expression were not observed in co-culture of mouse MSCs and rat islets (Fig. 2-a, b). These results indicate that nuclei of both MSCs and islet cells were mutually reprogrammed by one another in fusion cells.

### Islet Cell Apoptosis

**Figure 6:**
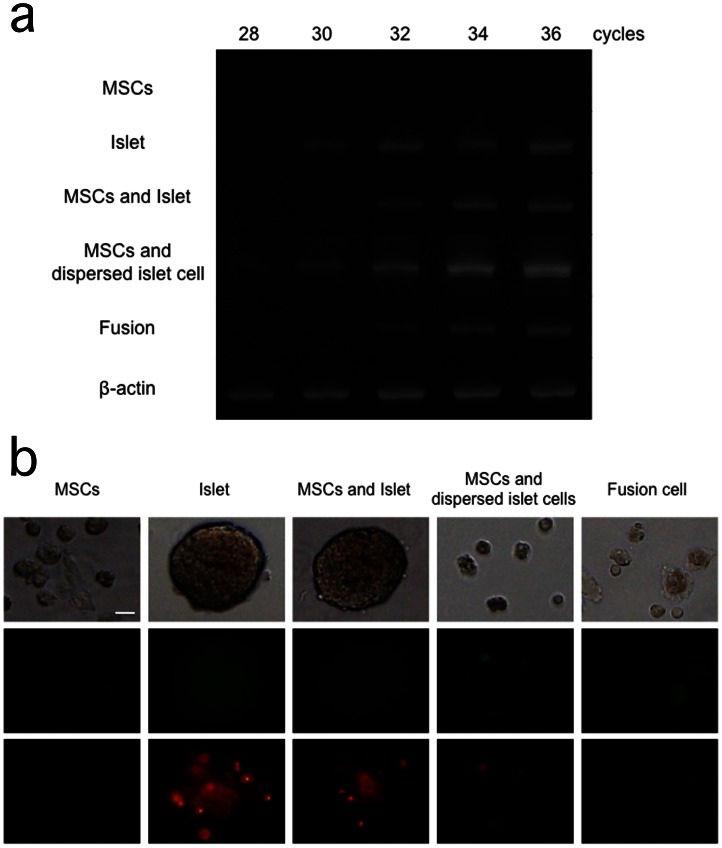
a: Caspase3 gene expression for each group by 28, 30, 32, 34 and 36 PCR cycles. b: Annexin staining for each group. Upper panels show light microscopic view. Middle panels show annexin staining (green: cell membrane), and lower panels show propidium iodide staining (red: nuclear). Scale bar: 20 µm.

Gene expression of caspase3 was detected in all groups except MSCs. PCR product was readily detected after 28 cycles in islet group and MSCs and dispersed islet cells group. On the other hand, in MSCs and islet group and fusion group, it was detected after 30 cycles. All groups reached plateau by 36 cycles (Fig. 6-a). Therefore, caspase3 expression was reduced in fusion cells in comparison to that in co-culture of MSCs and dispersed islet cells.

Annexin and PI positive cells were scarcely detected in MSCs. However, in other groups (Islet, MSCs and islet, MSCs and dispersed islet cell, fusion cell), they were observed after 1-day culture. Co-culture of MSCs with islets appeared to reduce annexin and PI positive cells in comparison to islet alone. Annexin and PI positive cells appeared to be reduced in fusion cells in comparison to co-culture of MSCs and dispersed islet cells (Fig. 6-b).

### Islet Cell Proliferation

**Figure 7:**
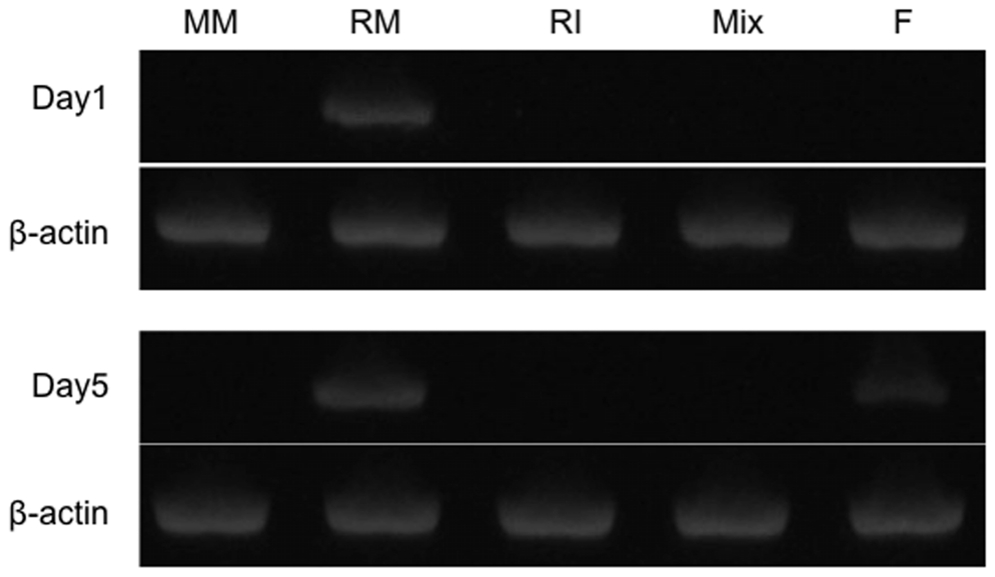
Rat Ki-67 gene expression of each group by RT-PCR. MM: mouse MSCs, RM: rat MSCs, RI: rat islets, Mix: co-culture of mouse MSCs with rat islets (non-fused) and F: Fusion cells between mouse MSCs and rat islet cells.

On day 1, only rat MSCs expressed rat Ki-67. However, on day 5, fusion cells newly expressed rat Ki-67. Ki-67 expression was not detected in co-culture of mouse MSCs and rat islet (Fig. 7). This result suggests that rat islet nuclei obtained proliferative capability after cell fusion with mouse MSCs.

### *In vivo* Transplantation

**Figure 8:**
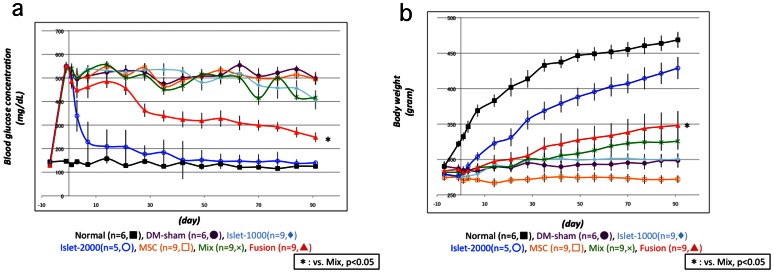
Results of blood glucose (a) and body weight (b) in *in vivo* transplantation study. Group 1(Normal control, n = 6,▪), Group 2(DM-sham, n = 6,•), Group 3(Islet-1000, n = 9,♦), Group 4(Islet-2000, n = 5,○), Group 5(MSCs, n = 9,□), Group 6(Mix, n = 9,×), Group 7(Fusion, n = 9,▴). *: p<0.05 vs Group 6.

Effect of renal subcapsular transplantation of fusion cells was examined in seven groups as follows: Group1; Normal control (n = 6), Group 2; sham operated streptozotocin-induced DM rats (n = 6), Group 3; sub-optimal islet mass (1000 rat islets: n = 9), Group 4; optimal islet mass (2000 islets: n = 5), Group 5; MSCs alone (1×10^6^ cells: n = 9), Group 6; Non-fused cell mix of dispersed islet cells from 1000 islets and MSCs 1×10^6^ cells (n = 9), Group 7; Fusion cell processed from dispersed cells from 1000 islets and MSCs 1×10^6^ cells (n = 9). Figures 8-a and b show changes in blood glucose (BG) and body weight, respectively. In group 4 (2000 islets), BG was decreased promptly and hyperglycemia was corrected to the normal range ever since POD 40. In the same group, body weight also showed a significant increase with a slope similar to the control group. There were no significant parametric changes either in BG or body weight in group 3 (1000 islets). Transplantation of MSCs alone in group 5 did not show any effect on BG or body weight in comparison to DM control of group 2. In group 6 (non-fused cell), BG did not decrease less than 400 mg/mL and body weight was slightly increased. Group 7 (fusion cell) showed continuous decrease in BG during the observation period and a greater increase in body weight compared to group 6 was observed. A significant difference was detected between group 7 and group 6 both in BG and body weight as well as between group 7 and group 3 (p<0.05, Fig. 8-a, b).

## Discussion

In this study, fusion cells between islet cells and MSCs were successfully prepared by electrofusion as shown in Figures 3 and 4. The fusion cells maintain sustained β-cell function *in vitro* and ameliorate hyperglycemia in a progressively increasing manner, suggesting potential clinical use of these fusion cells for diabetes therapy. In the literature, Barrera-Escorcia E, et al. have reported electrofusion of donor islet cells with poorly characterized dermal cells of allogeneic recipient in rats. They transplanted the fusion cells to examine the modulation of immunogenicity without *in vitro* characterization [32]. Although a slight improvement was seen in several parameters including blood glucose levels, they failed to obtain the reversion of the diabetic profile with transplantation of their fusion cells.

Artificial cell fusion was first enabled through the discovery of Sendai virus by Okada Y, et al. [33]. Then, polyethylene glycol method [34] and electrofusion [35] were put to practical use. Among these methods, electrofusion appears to be the most efficient in order to prepare large number of cells necessary for transplantation therapy. In addition, electrofusion is suitable for clinical used because it does not need special chemicals or biological materials that may affect the safety of the processed cells.

In the literature, Soleimani M et al. [36] reported a protocol for isolation and culture of MSCs from mouse bone marrow using primary culture with frequent medium changes, suggesting that MSCs can be obtained without positive or negative selection. Many other researchers use the similar protocols without cellular selection to obtain MSCs. In the present study, bone marrow-derived cells show morphology similar to typical MSCs and expressed several MSC-specific markers, i.e., CD73 (also known as ecto-5′-nucleotidase, differentiation marker of lymphocytes), CD105 (also known as Endoglin, a component of TGF-beta receptor), sca-1 (also known as Ly-6A, expressed in hematopoietic stem cells, skeletal muscle cells, epithelial stem cells, lymphocytes and macrophages) and CD106 (also known as VCAM-1, expression is strongly downregulated in MSCs after differentiation to adipo-, osteo-, and chondrocytes) [37]–[38]. Expression of these markers is known to be involved in the minimal criteria for defining multipotent MSCs [39]. On the other hand, as to the negative markers, CD34 and CD45, that are well known as hematopoietic stem cells markers, were positive in original bone marrow cells but were not detected in MSCs. Lack expression of these markers is also involved in the minimal criteria [39]. From these results, we consider that our bone marrow-derived cells used in the present study were consistent with putative MSCs.

In the present in vitro study, co-culture of islet cells with MSCs enhanced sustainability of β-cell function to certain extent and cell fusion of islet cells and MSCs could further enhanced it. The protective effect of MSCs on islets was previously reported [22] and our co-culture study confirmed this. Additionally, the present study further showed that cell fusion of islet cells with MSCs dramatically enhances sustainability of β-cell function *in vitro*.

Present RT-PCR examination for reprogramming clearly showed that nuclei of both islet cells and MSCs were mutually reprogrammed by electrofusion. Therefore, these fusion cells can be considered as special cells with β-cell function and robustness of MSCs. In fact, we observed sustained insulin secretion during the experimental period *in vitro* and this seems to be caused by the MSCs’ antiapoptotic nature. On the other hand, although the protective effect of MSCs on co-cultured islets is often attributed to signaling molecules and cytokines released from MSCs [22], co-culture of MSCs with islet cells did not cause reprogramming of islet cell nuclei (Fugure 5-a, b).

Palermo A. et al. [40] have reported nuclear reprogramming in fusion cells of human keratinocytes and mouse muscle cells and found that extensive changes were observed within 4 days. They also reported that, depending on the ratio of the cell number of two cell types, either phenotype could be dominant. In the present reprogramming study, we examined only one ratio of 1,000 islets and 1×10^6^ MSCs, that can be estimated approximately 1∶1 cell ratio at only one time point, after an over night culture. Apparently, the influence of cell ratio and time point awaits further investigation.

As to the anti-apoptotic nature of MSCs, caspase3 gene expression was decreased in co-culture of MSCs with islets in comparison to islet alone (Fig. 6-a), suggesting anti-apoptotic effect of MSCs on islets. MSCs, however, did not show the effect when co-cultured with dispersed islet cells in which apoptotic tendency is thought to be increased (Fig. 6-a). Finally, caspase3 gene expression was decreased in fusion cells between MSCs and dispersed islet cells (Fig. 6-a). These results indicate that electrofusion of MSCs can inhibit apoptotsis of islet cell nuclei even after they are dispersed.

In the present *in vivo* transplantation study, sub-optimal number of islet cells fused with MSCs gradually normalized the blood glucose levels in weeks. This suggests that the β-cell function of transplanted fusion cells was gradually enhanced. Some other studies have reported that co-transplantation of islets with MSCs could facilitate engraftment of the islets [21]. However, in the present study, we have failed to show this in the group 6 of non-fused islet cells and MSCs co-transplantation, probably because islet mass of 1,000 islets was too small to show the effect even transplanted with MSCs. On the other hand, fusion cells between islet cells and MSCs showed significant effect of transplantation in comparison to the group 3 (sub-optimal islet number) and even to the group 6 (co-tra*nsplantation with MSCs). These results indicate that cell fusion with MSCs provides a more potent facilitative effect on islet transplantation than co-transplantation of MSCs.

The mechanism of this observation remains to be elucidated. But, from present *in vitro* study, one possibility is sustainability of β-cell function that overcomes glucose toxicity caused by hyperglycemia. Another possibility is proliferation of the fusion cells that exert β-cell function. Although it is not quantitatively analyzed in the present study, cell number of fusion cells appeared to increase during culture period as shown in Fig. 5-d: E, J and O. Furthermore, new expression of rat Ki-67 in fusion cells suggests that islet cell nuclei obtain proliferation capability at least 5 days after cell fusion (Fig. 7). Therefore, fusion cells between islet cells and MSCs seem durable and proliferative to exert increasing β-cell function after transplantation.

Possible relationship between carcinogenesis and interaction or cell fusion of bone marrow-derived cells is suggested [41]. Although we did not find any tumor formation at the transplantation site of fusion cells at POD 91, tumorigenicity of fusion cells should be carefully addressed in the future studies.

In conclusion, this study showed that electrofusion of islet cells with MSCs is an efficient method to obtain potent and robust insulin-secreting cells that can potentially have a clinical interest since it could help to reduce the number of islet cells needed to achieved a therapeutic benefit in diabetic patients.
